# Pyrimidine synthase CAD deamidates and inactivates p53

**DOI:** 10.1038/s41422-025-01112-9

**Published:** 2025-04-17

**Authors:** Yue Qi, Zizheng Tan, Hanyu Chen, Ziqi Xiao, Liang Zhang, Boxuan Wu, Chennan Liu, Yunqian Gao, Xueyan Yang, Lingqian Wu, Lei Lu, Hongyan Wang

**Affiliations:** 1 Obstetrics and Gynecology Hospital, State Key Laboratory of Genetic Engineering, School of Life Sciences, Children’s Hospital, Fudan University, Shanghai, China; 2 Shanghai Key Laboratory of Metabolic Remodeling and Health, Institute of Metabolism and Integrative Biology, Fudan University, Shanghai, China; 3 Pediatric Translational Medicine Institute, Shanghai Children’s Medical Center, School of Medicine, Shanghai Jiao Tong University, Shanghai, China; 4 The Center for Medical Genetics, Hunan Key Laboratory of Medical Genetics & Hunan Key Laboratory of Animal Models for Human Diseases, School of Life Sciences, Central South University, Changsha, Hunan China; 5 Prenatal Diagnosis Center of Shenzhen Maternity & Child Healthcare Hospital, Shenzhen, Guangzhou China

**Keywords:** Cell biology, Post-translational modifications

Dear Editor,

p53, a well-known important tumor suppressor, is central for regulating cell cycle in response to DNA damage and altered metabolism.^1^ With a short half-life, p53’s stability and activity are primarily controlled by post-translational modifications (PTMs).^2^ Deamidation, a modification catalyzed by metabolic enzymes with glutamine amidotransferase (GAT) activity, converts residues asparagine or glutamine into aspartate or glutamate, resulting in a more negative shift of the charge and the subsequent protein function alteration.^3,4^ Whether and how deamidation regulates the activity of p53 remains unclear in vivo. 6-diazo-5-oxo-L-norleucine (DON) is a glutamine analog that inhibits glutamine metabolism by competitively inhibiting multiple glutamine-dependent enzymes, including the Carbamoyl-phosphate synthetase 2, Aspartate transcarbamoylase, and Dihydroorotase (CAD), a vital rate-limiting metabolic enzyme in pyrimidine biosynthesis.^5,6^ DON inhibits glutamine metabolism and acts as a broad-spectrum deamidase inhibitor, yet its non-metabolic functions in cancer therapy have been largely overlooked in prior studies.

Here we aimed at identifying alternative regulatory targets of DON beyond the glutamine metabolic pathway. We observed a significant decrease in cell numbers concurrent with the induction of cell cycle arrest and apoptosis in DON-treated cells compared to the DMSO control (Fig. 1a; Supplementary information, Fig. S1a–c). To investigate the molecular determinants, we conducted RNA-sequencing (RNA-seq) experiments. The differentially upregulated genes in the DON treatment group were significantly enriched in the p53 signaling pathway (Fig. 1b, c), which was further confirmed by quantitative reverse transcription PCR (RT-qPCR) and western blotting assays in multiple cell lines (Fig. 1d; Supplementary information, Fig. S1d). To ascertain whether DON suppresses cell proliferation primarily through the p53 signaling pathway, we generated a *p53*^−/−^ HCT116 cell line. While DON treatment significantly inhibited cell growth in p53 wild-type (WT) cells, its inhibitory effect was markedly attenuated in p53-deficient cells (Fig. 1e). In *p53*^−/−^ cells, p53 downstream target genes, including *p21*, *PUMA* and *BAX* no longer responded to DON treatment (Fig. 1f). Taken together, these findings demonstrated that DON suppresses cell proliferation primarily depending on the activation of p53 signaling pathway.

**Fig. 1:**
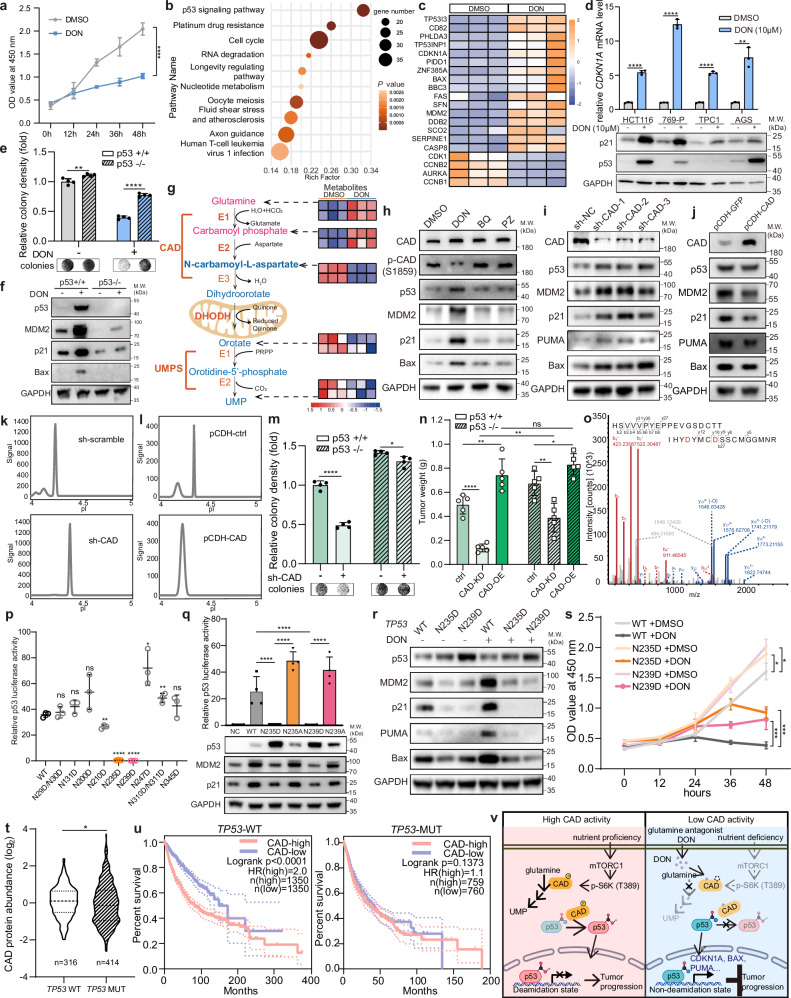
DON activates the p53 signaling pathway by inhibiting p53 deamidation, which is mediated by the pyrimidine synthase CAD, thereby suppressing tumor cell growth. **a** Relative growth of HCT116 cells at the indicated time points following either DON (10 μM) or DMSO treatment. Data are expressed as mean ± SD, *n* = 3, one-way ANOVA followed by Tukey. **b** KEGG enrichment analysis of RNA-seq results for p53 downstream target genes in HCT116 cells. Red corresponds to high expression and blue corresponds to low expression. Cells were subjected to vehicle (DMSO) or DON (10 μM) treatment for 24 h. **c** Heatmap showing RNA-seq results for p53 downstream target genes in HCT116 cells. Red indicates high expression and blue indicates low expression. Cells were subjected to vehicle (DMSO) or DON (10 μM) treatment for 24 h. **d** RT-qPCR analysis (top panel) of relative mRNA levels of *CDKN1A* in HCT116, 769-P, TPC1, AGS cells and western blotting analysis (bottom panel) of p21 and p53 levels in the 4 cell types treated with DMSO or DON (10 μM) for 24 h. Data are expressed as mean ± SD, *n* = 3, Student’s *t*-test. **e** Colony formation assay of HCT116 *p53*^+/+^ and *p53*^−/−^ cells treated with DMSO or DON (10 μM). Data are expressed as mean ± SD, *n* = 4 Student’s *t*-test. **f** Western blotting analysis of the expression of p53 and its targeted genes in HCT116 *p53*^−/−^ cells. **g** Schematic of the de novo pyrimidine synthesis pathway (left panel) and the indicated metabolite levels, measured by LC-MS/MS, in TPC1 (first row for each metabolite) and HCT116 cells (second row for each metabolite) under treatment with DMSO or DON (10 μM). **h** Western blotting analysis of whole-cell lysates of HEK293 cells treated with DMSO, DON (10 μM), BQ (10 μM) and PZ (10 μM) for 12 h. **i** Western blotting analysis of whole-cell lysates in control (sh-scramble) or CAD-knockdown (sh-CAD) HCT116 cells. **j** Western blotting analysis of whole-cell lysates in control (pCDH-GFP) or stable CAD-overexpression (pCDH-CAD) HCT116 cells. **k** Capillary electrophoresis-based charge separation and immunoblot of p53 in control (sh-scramble) and CAD-knockdown (sh-CAD) HEK293 cells. **l** Capillary electrophoresis-based charge separation and immunoblot of p53 in control (pCDH-ctrl) or stable CAD-overexpression (pCDH-CAD) HEK293 cells. **m** Colony formation assay of HCT116 WT cells and HCT116 *p53*^−/−^ cells with or without CAD depletion. Data are expressed as mean ± SD, *n* = 4, Student’s *t*-test. **n** Statistical analysis of xenograft models showing in vivo tumor growth injected with HCT116 cells with or without p53, and with manipulated CAD expression. The tumors were measured 21 days after injection. Data are expressed as mean ± SD, *n* = 5, two-way ANOVA followed by Tukey. **o** The *m*/*z* spectra of the peptide containing N235D and N239D is shown, with D marked in red due to deamidation, of the p53-Flag protein purified with Flag-coated magnetic beads from HCT116 cells. **p** Dual luciferase reporter assay of p53 from HeLa cells transfected with the indicated mutant p53 plasmids. Data are expressed as mean ± SD, *n* = 3, one-way ANOVA followed by Tukey. **q** Dual luciferase reporter assay (upper) and western blotting assay (lower) of p53 from HeLa cells transfected with the indicated mutant p53 plasmids. Data are expressed as mean ± SD, *n* = 4, with *P* values calculated by one-way ANOVA, followed by Tukey. **r** Western blotting analysis of the expression of p53 and its targeted genes in WT, and p53-N235D, p53-N239D endogenous mutant TPC1 cells. **s** Relative growth of WT and p53-N235D, p53-N239D endogenous mutant TPC1 cells under treatment of DMSO or DON (10 μM) using CCK8 assay. Data are expressed as mean ± SD, *n* = 3, one-way ANOVA followed by Tukey. **t** Analysis of the genomic and proteomic data from the Clinical Proteomic Tumor Analysis Consortium (CPTAC) comparing the protein levels of CAD between patients carrying WT *TP53* with those carrying mutant *TP53* in cohorts including Breast invasive carcinoma (BRCA), Colon adenocarcinoma (COAD), Glioblastoma multiforme (GBM), Head and Neck squamous cell carcinoma (HNSCC), Lung adenocarcinoma (LUAD), Prostate adenocarcinoma (PDAC) and Uterine Corpus Endometrial Carcinoma (UCEC). Welch’ s *t*-test. **u** Analysis of TCGA Pan-Cancer data comparing overall survival curves between patients carrying WT *TP53* (left panel) and those with mutant *TP53* (right panel), stratified by high and low CAD expression quartiles. Log-rank test. **v** A schematic model of CAD-mediated p53 deamidation. *P < 0.05; **P < 0.01; ***P < 0.001; ****P < 0.0001; ns, no significance.

To investigate the regulatory targets of DON, we conducted intracellular targeted metabolomic analysis of HCT116 cells treated with or without DON. Enrichment analysis revealed significant changes in metabolites linked to pyrimidine synthesis (Supplementary information, Fig. S2a). The de novo pyrimidine biosynthesis pathway, essential for DNA and RNA synthesis, begins with the formation of carbamoyl phosphate from glutamine and hydrogen carbonate, followed by its sequential conversion into N-carbamoyl-L-aspartate and then dihydroorotate, catalyzed by the CAD. Dihydroorotate dehydrogenase (DHODH) subsequently converts dihydroorotate into orotate, which is further processed by uridine 5’-monophosphate synthase (UMPS) to produce uridine monophosphate (UMP), the precursor for pyrimidine synthesis.^7^ After 24 h of DON treatment, N-carbamoyl-L-aspartate, orotate and UMP levels were reduced, while glutamine and carbamoyl phosphate were accumulated (Fig. 1g), which suggests that DON suppresses the activity of CAD. To investigate whether disruption of de novo pyrimidine biosynthesis activates p53 signaling, we inhibited DHODH and UMPS using brequinar (BQ) and pyrazofurin (PZ), respectively. After 12 h, p53 target genes were significantly activated by DON, while BQ and PZ elicited only a slight effect. UMP levels decreased in all three groups (Fig. 1h; Supplementary information, Fig. S2b). Furthermore, DON-treated cells showed a significant reduction in UMP levels 4 h after treatment, whereas the mRNA level of *CDKN1A*, a known target of p53, was already upregulated after just 2 h (Supplementary information, Fig. S2c). N-carbamoyl-L-aspartate was the most significantly decreased metabolite, whereas geranylgeranyl pyrophosphate (Geranyl-PP), a key metabolite in protein geranylgeranylation, demonstrated the highest increase after DON treatment (Supplementary information, Fig. S2d). However, these intermediate metabolites did not affect the expression of p53 or its downstream target genes (Supplementary information, Fig. S2e, f). DON treatment significantly activated the p53 pathway, even when glutamine was removed from the cultured medium (Supplementary information, Fig. S2g). These experiments ruled out the possibility that significant alterations in other metabolites affected by DON or the inhibition of pyrimidine synthesis was responsible for p53 activation. These data suggest that DON’s regulation of p53 activity is independent of pyrimidine metabolism disruption by CAD inhibition.

CAD is a multifunctional enzyme involved in the initial three rate-limiting steps of de novo pyrimidine synthesis and is closely linked to cell cycle progression.^8^ Elevated CAD expression correlates with tumor progression and recurrence in prostate cancer and hepatocellular carcinoma, and amplification of the *CAD* genes has been reported in various human tumors, making it a potential prognostic marker.^9^ To investigate whether CAD directly regulates p53 activity, we generated stable CAD-knockdown and CAD-overexpression HCT116 cells. Western blotting assays showed that CAD depletion upregulated p53 and its downstream targets, while CAD overexpression suppressed p53 activity (Fig. 1i, j). Given the non-metabolic deamidase activity of CAD and the deamidase inhibitor role of DON,^10^ we hypothesized that CAD may regulate p53 activity through deamidation. As deamidation exclusively occurs at asparagine or glutamine residues, there is a resulting shift of the protein charge toward a more negative state. To investigate whether DON prevents deamidation of p53, we evaluated the charge status of p53 in the presence and absence of DON treatment using capillary electrophoresis-based charge separation and immunoblotting with a Nanopro 1000 system. p53 protein exhibited a more negative charge in the control samples, which indicates the deamidation status of p53, in contrast to the more neutral charge state found in DON-treated proteins (Supplementary information, Fig. S3a). Charge analysis also revealed that CAD overexpression shifted the charge of p53 towards the negative state and CAD depletion had the opposite effect (Fig. 1k, l). Serum-activated mTORC1 signaling stimulates CAD phosphorylation at Ser1859 via S6 kinase, enhancing CAD activity.^11,12^ This could have subsequently led to p53 inactivation (Supplementary information, Fig. S3b, c), suggesting that nutritional factors promote p53 deamidation and suppression of its target genes through CAD phosphorylation. To rule out the involvement of other deamidases in p53 inactivation, we individually knocked down different deamidases in HCT116 cells. Only CAD knockdown significantly increased p53 activity, as shown by a luciferase reporter assay (Supplementary information, Fig. S3d). Co-immunoprecipitation in HEK293 cells confirmed the interaction between p53 and CAD, with p53 binding to CAD’s CPS-B and GAT domains (Supplementary information, Fig. S3e–i).

Next, we investigated the impact of CAD on p53-mediated cell proliferation. CAD depletion significantly slowed the growth of *p53*^+/+^ cells, but had only a minimal effect on *p53*^−/−^ cells (Fig. 1m). Consistently in the xenograft models, DON significantly inhibited tumor growth in p53 WT tumors, with a more limited effect in p53-null tumors (Supplementary information, Fig. S3j). CAD depletion had a less significant impact on tumor growth in p53-null cells. While CAD overexpression significantly promoted tumor growth in p53 WT cells, the effect remained equal in p53-null cells (Fig. 1n; Supplementary information, Fig. S3k). These findings indicate that DON more effectively inhibits tumor growth in the presence of WT p53 protein and high levels of CAD expression. In addition, p53 pathway was significantly upregulated by the CAD-specific inhibitor 2-TCPA (Supplementary information, Fig. S3m).^13^ Using liquid chromatography-tandem mass spectrometry (LC-MS), we identified the deamidation of eight asparagine residues, which were converted to aspartate within p53 (Fig. 1o; Supplementary information, Fig. S4a–f). The in vitro deamidation assay demonstrated the enzymatic activity of CAD in deamidating the p53 protein (Supplementary information, Fig. S5a–d). To explore the impact of deamidation on p53 function, we generated mutations of residues asparagine to aspartate to mimic the deamidated state. Results from dual luciferase reporter and western blotting assays revealed that the N235D and N239D mutations almost completely abolished the transcriptional activity of p53 on its targeted genes (Fig. 1p; Supplementary information, Fig. S6a). Converting the two residues, N235 and N239, to alanine — rendering them incapable of undergoing deamidation — sustained and even amplified the function of p53 compared to its WT form (Fig. 1q). CRISPR/Cas9-generated TPC1 cell lines with endogenous N235D or N239D mutations further confirmed these findings, showing significant loss of p53 activity and resistance to DON treatment (Fig. 1r; Supplementary information, Fig. S6b). These mutant cells proliferated faster than WT cells and showed reduced sensitivity to DON’s inhibitory effects (Fig. 1s). Together, deamidation at N235 and N239 significantly impaired p53’s signaling activity, thereby promoting tumor cell proliferation.

To assess the clinical impact of CAD overexpression on tumor growth based on p53 status, we analyzed data from CPTAC, including BRCA, COAD, GBM, HNSCC, LUAD, PDAC and UCEC. We found significantly higher CAD protein levels in patients with WT-*TP53* compared to those with mutant *TP53* (Fig. 1t). Analysis of TCGA Pan-Cancer data revealed a significant negative correlation between CAD expression and survival in WT *TP53* patients, whereas this correlation was less distinct in mutant *TP53* patients (Fig. 1u). In tumor types such as kidney renal clear cell carcinoma, kidney renal papillary cell carcinoma, brain lower grade glioma, sarcoma, and skin cutaneous melanoma, CAD expression was higher than in normal healthy tissues and negatively correlated with overall survival in the p53 WT population. Adrenocortical carcinoma, despite not showing elevated CAD expression, still exhibited a similar negative correlation with survival (Supplementary information, Fig. S7a–g).

Our findings highlight deamidation as a new PTM of p53, essential for inhibiting the activity of p53. It becomes more reasonable for us to understand what indicated by an increase of *CAD* copy number or expression level of CAD in tumors. However, nucleotide pool imbalance caused by CAD inhibition or DON treatment may further amplify p53 activation through DNA damage response.^14^ Our study reveals that, beyond its role in pyrimidine biosynthesis, CAD deamidates and suppresses p53, thereby promoting tumor growth. High CAD expression in tumors may inactivate WT p53 through deamidation, contributing to tumor progression in a manner similar to p53 loss-of-function mutations. Thus, both high CAD expression and p53 mutations may represent evolutionary mechanisms that drive tumor development. The glutamine analog DON effectively inhibited CAD and restored p53 activity (Fig. 1v). Here we found that deamidation at N235/N239 significantly suppressed p53 transcriptional activity. Intriguingly, these residues reside within a global suppressor motif of p53, where other mutations (e.g., R/Y/K/W/F) have been shown to restore functionality in certain tumor-associated p53 mutants.^15^ This suggests that electrostatic modulation at N235 and N239 play a key role in regulating p53 function. Elucidating how these residues impact the global structure and function of the p53 protein deserves future investigation. Our study suggests a potential strategy for stratifying patients for cancer therapy, indicating that those with WT p53 and high CAD expression may benefit from treatment with glutamine antagonists.

## Supplementary information

Supplementary information

## Data Availability

The raw data for bulk RNA-seq of HCT116 cell samples treated with DMSO or DON have been deposited in GEO database under GSE286372.
