# Supplementary material for: Pyrimidine synthase CAD deamidates and inactivates p53
Source: Cell Res. 2025 Apr 17;35(7):520–3. doi: 10.1038/s41422-025-01112-9 (PMC12205080; doi:10.1038/s41422-025-01112-9)
Supplement: Supplementary file 1 — Supplementary information [file 41422_2025_1112_MOESM1_ESM.pdf]

Supplementary Materials for

Pyrimidine synthase CAD deamidates and inactivates p53

Yue Qi[1,#], Zizheng Tan[2,#], Hanyu Chen[1], Ziqi Xiao[2], Liang Zhang[1], Boxuan Wu[2], Chennan Liu[2], Yunqian Gao[3], Xueyan Yang[1], Lingqian Wu[4], Lei Lu[2,*] and Hongyan Wang[1,2,5,*]

[1]Obstetrics and Gynecology Hospital, State Key Laboratory of Genetic Engineering, School of Life Sciences, Children's Hospital, Fudan University, Shanghai 200011, China.

[2]Shanghai Key Laboratory of Metabolic Remodeling and Health, Institute of Metabolism and Integrative Biology, Fudan University, Shanghai 200438, China

[3]Pediatric Translational Medicine Institute, Shanghai Children's Medical Center, School of Medicine, Shanghai Jiao Tong University, Shanghai 200127, China.

[4]The Center for Medical Genetics, Hunan Key Laboratory of Medical Genetics & Hunan Key Laboratory of Animal Models for Human Diseases, School of Life Sciences, Central South University, Changsha 410078 China

[5]Prenatal Diagnosis Center of Shenzhen Maternity & Child Healthcare Hospital, Shenzhen 518028, China

[#]These authors contributed equally.

*Correspondence to: wanghy@fudan.edu.cn and lulei@fudan.edu.cn

This PDF file includes:

Materials and Methods

Extended results and discussion

Reference

Supplementary Text

Figures S1 to S7

Figures S1 DON activates p53 signaling

Figures S2 Significant alterations of metabolites after DON treatment inefficiently activate p53

Figures S3 CAD deamidates p53

Figures S4 Deamidation residues within p53

Figures S5 In vitro deamidation assay of p53 by CAD

Figures S6 Deamidation at the conserved N235 or N239 residue of p53 impairs its transcriptional activity

Figures S7 Survival analysis of CAD expression between different TP53 status in different tumor types

Table S1 List of oligos in this study

Materials and Methods

Cell culture

HeLa, HCT116, AGS, TPC1, A549, and U2OS cell lines were cultured in DMEM medium supplemented with 10% (v/v) fetal bovine serum and 1% penicillin/streptomycin. The 769-P cell line was maintained in RPMI 1640 medium containing 10% (v/v) fetal bovine serum and 1% penicillin/streptomycin. TP53 gene knock-out or knock-in in HCT116 and TPC1 cells were achieved via CRISPR-Cas9 transfection of px330-mcherry plasmid loaded with specific sgRNA sequences and pcDNA3.1 plasmid loaded with mutated donor sequences, followed by fluorescence-activated cell sorting. 24 hours prior to harvesting, DON was added to the cells at a concentration of 10 μg/ml by dilution in the medium. All cells were incubated in a humidified incubator at 37°C with 5% CO_2.

Immunoblotting and immunoprecipitation

Protein samples were extracted through cell lysis with the use of an ice-cold lysis buffer (20 mM Tris at pH 7.5, 150 mM NaCl, 1% Triton X-100, 10 mM β-glycerophosphate, 0.5% sodium pyrophosphate, 1 mM EDTA, 1 mM DTT, and 1 mM Na_3VO_4 supplemented with protease inhibitor cocktail (4693132001; Roche) and phosphatase inhibitors (4906845001; Roche). The lysates were incubated on ice for 20 min and subjected to centrifugation at 4°C at 12000 rpm for 10 min. The supernatant was then mixed with 5× protein loading buffer (C508320; Sangon Biotech) and boiled. Immunoprecipitation assays were conducted by incubating the resultant supernatant overnight with pre-washed magnetic beads conjugated with anti-FLAG (M8823; Sigma-Aldrich) or anti-HA antibody (88836; Thermo Scientific) in a rotating incubator at 4°C. The protein samples were subjected to 4-20% SurePAGE™ Bis-Tris gradient gels (M00655; Genscript) before transfer to PVDF membrane (ISEQ00010; millipore). Subsequently, the membrane was incubated with the indicated primary antibodies

overnight at 4°C, and then with secondary antibodies for 1 h before performing immunoblotting.

In vitro deamidation assay

293T cells were transfected with either: (1) pcDNA3.1-FLAG empty vector (negative control), (2) wild-type (WT) CAD-FLAG construct, or (3) catalytic mutant C252S CAD-FLAG construct using lipo8000 (C0533, Beyotime). Forty-eight hours post-transfection, cells were lysed in cell lysis buffer containing protease inhibitor cocktail (Roche cOmplete™). FLAG-tagged proteins were immunoprecipitated using anti-FLAG M2 magnetic beads through overnight incubation at 4°C with rotation. Beads were washed three times with ice-cold lysis buffer followed by equilibration in reaction buffer (PBS with 1× protease inhibitors). Recombinant human p53 protein (N-terminal 6xHis-SUMO-tagged, CSB-EP024077HU, CUSABIO) was diluted in 200 μL PBS containing protease inhibitor cocktail. Equal aliquots (200 μL) were incubated with each bead-bound CAD variant for 3 h at room temperature. Reactions were terminated by adding 5× loading buffer (62.5 mM Tris-HCl pH 6.8, 2% SDS, 10% glycerol) and denaturing at 95°C for 10 min. Eluted proteins were subjected to LC-MS/MS analysis.

Cell proliferation assay

In order to evaluate cell proliferation, a CCK-8 assay was carried out in accordance with the manufacturer's instructions (A311-01; Vazyme Biotech). Specifically, HCT116 and TPC1 wild-type (WT), CAD-overexpression, and CAD-knockdown cells were seeded at 1×10^4 cells per well in a 96-well plate and exposed to relevant experimental conditions for 24 hours. CCK-8 reagent was added to the wells, and the plate was incubated for 1 hour at 37°C in the dark at the indicated time points. Absorbance was subsequently quantified at 450 nm using a microplate reader, and the cell proliferation rate was determined after background value subtraction. Experiments were conducted in triplicate and repeated at least three times independently to ensure reproducibility of the results.

Quantitative RT-PCR

Total RNA was isolated from cells utilizing RNA isolater Total RNA Extraction Reagent (R401; Vazyme Biotech). The first strand cDNA was transcribed from 1μg total RNA using HiScript III RT supermix for qPCR (+gDNA wiper) (R323; Vazyme Biotech) using the guidelines provided by the manufacturer. Quantitative real-time reverse transcription PCR was subsequently performed using ChamQ Universal SYBR qPCR Master Mix (Q711; Vazyme Biotech) on the QuantStudio™ 1 Real-Time PCR System (Applied Biosystems). The relative levels of mRNA were standardized relative to beta-actin concentrations. A table containing primer sequences is provided in Table S1.

Luciferase assay

HCT116 and H1299 cells were seeded into 24-well plates at an optimal density of 30%-40% with four replications per group, and maintained incubation for no less than 12 h. Plasmids containing 500 ng of p53-WT or p53-MUT expressing plasmid, 100 ng luciferase reporter plasmid, and 10 ng Renilla plasmid were transfected using lipofectamine 2000 reagent (11668019; Invitrogen). The transfected cells were sustained for at least 24 hours before they were lysed, and the luminescent signals were estimated by conforming to the manufacturer's specifications using the Dual-Luciferase Reporter Assay System (Promega product code: E1960). The relative luciferase activity was then normalized against the Renilla activity of each sample to obtain accurate measurements.

Glutaraldehyde fixation assay

The Glutaraldehyde fixation assay was conducted following the published methodology [1]. The HeLa cells were first transfected with p53-WT or p53-MUT plasmids for over 24 h, and subsequently lysed using the lysis buffer as described in the methodology of Immunoblotting and Immunoprecipitation. The resulting supernatants were then collected into pre-cooled tubes. Glutaraldehyde was appropriately diluted to 2.5%, and added to the cell lysates to achieve a final concentration of 0.01%, after

which the whole mixture was incubated under room temperature for 20 minutes. The lysates were then boiled with 2x loading buffer for 10 minutes and subjected to an immunoblotting assay. The following antibodies were used: phospho-p53 (S15) antibody (CST, 9284), p53 antibody (CST, 9282), p53 antibody (DO-1) (Santa Cruz Biotechnology, sc-126), Anti-MDM2 antibody (2A10) (abcam, ab16895), p21 Waf1/Cip1 (12D1) Rabbit mAb (CST, 2947), PUMAα/β antibody (G-3) (Santa Cruz Biotechnology, sc-374223), Bax antibody (CST, 2772), GAPDH mouse (ABclonal, AC002), CAD antibody (CST, 11933), Phospho-CAD (Ser1859) Antibody (CST, 12662), p70 S6 Kinase (49D7) Rabbit mAb (CST, 2708), Phospho-p70 S6 Kinase (Thr389) Antibody (CST, 9205), HA-tag-Rabbit (CST, 3724), Flag-tag rabbit mAb (CST, 14793).

Immunofluorescence

Cells were seeded onto pre-coated coverslips with gelatin in 24-well plates at a density of 30%. Following transfection with specific plasmids or indicated treatment for 24 hours, cells were fixed with 4% paraformaldehyde (PFA) for 15 minutes at room temperature and subsequently washed with PBS three times. Permeabilization of the cells was achieved with 0.2% Triton X-100 for 10 minutes, followed by another three washes in PBS. Next, cells were blocked for one hour at room temperature by incubation in 3% BSA solution dissolved in PBS. Subsequently, the coverslips were removed from the plate, placed on a parafilm-covered glass slide, and incubated with the specific primary antibodies diluted with blocking buffer at 4°C overnight. The next day, the primary antibodies were removed and the cells were washed three times with PBST (PBS containing 0.1% Tween-20) prior to the addition of secondary antibodies for a one-hour incubation at room temperature. Finally, cells were incubated with 4',6-diamidino-2-phenylindole (DAPI) for 10 minutes at room temperature and followed by three washes in PBST. Coverslips were then placed on new glass slides with added fluorescence mounting medium prior to acquiring fluorescence images on confocal microscope (Zeiss LSM 880).

Gene editing by CRISPR/Cas9

The TPC1 cell line was subjected to TP53 gene editing using a pX330-mcherry plasmid which is capable of expressing both Cas9 protein and a sgRNA using the CRISPR/Cas9 technique. The sgRNA sequences of interest can be found in Table S1. For CRISPR/Cas9-mediated homology-directed repair, a donor consisting of a double-stranded DNA molecule with a single nucleotide substitution of 600 bp in T-easy vector was generated. The TPC1 cells were co-transfected with the pX330-mcherry plasmids and the donor constructs at a ratio of 1:3. After being incubated for at least 48 h, mcherry fluorescence-positive cells were sorted into 96-well plates by BD FACS Aria cell sorter, with one cell per well. After a further two-week incubation, Sanger sequencing was performed to determine the genotypes of the cells and the successfully edited cells were used for subsequent experiments.

Generation of stable cell lines

To knock down the expression of the CAD gene, shRNA sequences targeting different regions of the gene were designed, as shown in Table S1. The selected shRNA sequences were cloned into pLKO.1-puro lentiviral vector. To establish stable cell lines overexpressing CAD, a lentiviral vector (pCDH-CMV-EF1-copGFP) carrying the CMV promoter, which drives the CAD gene, was constructed. Lentivirus particles were generated by co-transfecting the lentiviral vector with packaging plasmids pMDL, pRRE, pRSV-Rev, and envelope plasmid pMD2.G into HEK293T cells using lipofectamine 2000 reagent. After a medium change 12 hours post-transfection, lentiviral particles were collected following another 36 hours of incubation. The supernatants were filtered, and the resulting lentiviral particles were used to transduce target cells. Stable knock-down cell lines were selected with puromycin (1 µg/ml) for 5-7 days, while the stable over-expression cell lines were sorted into heterogeneous populations based on expression of GFP using flow cytometry. The CAD expression levels were subsequently analyzed using Western blotting.

Protein charge analysis by the NanoPro capillary-based immunoassay system[15]

To detect the isoelectric point (pI) of p53 protein at deamidated or non-deamidated states, we performed protein charge analysis using the NanoPro capillary-based immunoassay system which combines nanocapillary isoelectric focusing (cIEF) with UV-activated linking chemistry. Total protein assay was performed on an automated system for charge-based separation which maintained the native charge status [2]. Briefly, cells were lysed in Bicine/CHAPS lysis buffer (ProteinSimple; 040-764) and diluted to a final concentration of 0.1 mg/mL and then mixed with 1×Premix G2 pH 3-10 separation gradient (Protein simple; 040-968) and fluorescent pI standards (pI Standard Ladder 1) (Protein simple; 040-644). Samples, primary and secondary antibodies were diluted with antibody diluent (Protein simple; 040-309) to a final concentration of 1:50 and 1:100 respectively, and was loaded along with the chemiluminescent reagent (peroxide XDR and Luminol in a 1:1 ratio, Protein simple; 040-652) onto a 384-well plate. The plate was centrifuged at 2500 rpm for 5 min at 4°C to ensure no air bubbles remained in the well. Place the 384-well plate into the NanoPro system and the samples were separated based on their isoelectric points and cross-linked to the capillary wall using UV light followed by immunoprobing with anti-p53 and p-p53 (S15) antibodies. The signal was visualized by ECL and the digital image was analyzed through quantified peak area with Compass software (ProteinSimple).

RNA-sequencing

RNA-seq (library preparation and sequencing run) was performed at the APExBIO Technology facility. Total RNA was isolated using a TRIzol total RNA extraction kit (TIANGEN; Cat.No.DP424) and was quantified with NanoDrop, and equal amounts of RNA were used to generate cDNA library according to the manufacturer's instructions (Illumina,USA). Then the cDNA was amplified for the synthesis of the second chain of cDNA and the cDNA products were purified by AMPure XP system (Beckman Coulter, Beverly, USA). The library was sequenced using the Illumina NovaSeq 6000 sequencing platform (Paired end150) to generate raw reads. RNA-seq samples were mapped against the hg19 human genome assembly using HISAT2. Differentially expressed genes (DEGs) were identified by DEseq2. The clusterProfiler was used to

perform functional enrichment analysis for the annotated significant DEGs (p value<0.05) and KEGG pathway analysis. GSEA of the pre-ranked lists of genes by DESeq2 stat value was performed with the GSEA software.

Targeted tandem mass spectrometry (LC–MS/MS) for metabolomics

HCT116 and TPC1 cells were seeded into 6-well plates in triplicate. Cells were harvested at 70%-80% confluence after treated with DON (10 μM) for 24 h. LC-MS/MS of intracellular metabolites was prepared and performed as previously described [3]. Briefly, the cells were washed with PBS and then added with -80°C pre-frozen 80% (v/v) HPLC-grade methanol solution for metabolite extraction. After incubating the plates at -80°C for 20 min, cells were scraped and transferred to centrifuge tubes which were vortexed for 1 min. The extraction was incubated for another 30 min at -80°C. Then tubes were centrifuged at >4,000 g for 10 min (at 4-8°C) and the supernatant was transferred to new centrifuge tubes. Dry the samples to a pellet in 1.5 mL microcentrifuge tubes by SpeedVac/lyophilize or EVAP system, no heat. Metabolites were analyzed by LC-MS/MS using AB SCIEX ExionLCTM AD LC system coupled to a QTRAP 5500 (AB/SCIEX) mass spectrometer and the analysis and peak integration software OS-MQ Software (AB SCIEX). The differential metabolites between the control and treated groups were analyzed using the online tool MetaboAnalyst.

LC-MS analysis for protein post-translational modification detection and p53-binding proteins

Three 10 cm dishes of HCT116 and HEK293 cells were transfected with flag-tagged TP53 plasmids for 48 h. Cells were lysed with lysis buffer and the supernatants were added to the pre-washed anti-flag magnetic beads and rotated at 4°C overnight. The beads were washed with lysis buffer 3 times and the mixed with 5× SDS loading buffer and boiled for 10 min. The sample was subjected to SDS-PAGE and the gel was stained with Coomassie Brilliant Blue R-250 dye for 15 min and cleared for 30 min. The targeted band was excised from the gel followed by in-gel trypsin digestion. LC-MS

analysis was performed using a nanoflow EASYnLC 1200 system (Thermo Fisher Scientific, Odense, Denmark) coupled to an Orbitrap Exploris480 mass spectrometer (Thermo Fisher Scientific, Bremen, Germany). The results of were processed with UniProt human protein database and the using Protein Discoverer (version 2.4, thermo Fisher Scientific) with Mascot (version 2.7.0, Matrix Science). The mass tolerances were 10 ppm for precursor and fragment Mass Tolerance 0.05 Da. The deamidation on asparagine and glutamine were set as variable modifications.

Detection of cell cycle phases

Cells were treated with 10 μM of 5-ethynyl-2'-deoxyuridine (EdU) in culture media at 70-80% confluence 1 h before harvested. Cells were fixed with 4% polyformaldehyde for 15 min and washed with PBS containing 1% bovine serum albumin (BSA, Sangon biotechnology) at room temperature. Cells were permeabilized with 0.1% Triton X-100 in PBST for 10 min and then performed with Click-iT EdU labeling reaction (Thermofisher) in darkness at room temperature for 30 min. The components of Click-iT EdU labeling reaction mixture was freshly prepared as follows: 4 μL 100 mM copper sulfate pentahydrate $CuSO_4$, 0.2 μL 4 mM Alexa Fluor 488 azide, 20 μL 200 mg/mL ascorbate and 175.8 μL PBS for a total volume of 200 μL. The Alexa Fluor 488 labeled cells were washed with PBST and then incubated with 1 μg/mL FxCycle™ Far Red and 100ug/mL RNase A for DNA staining. The Alexa Fluor 488 and FxCycle™ Far Red double-labeled cells were then applied to the BD caliber flow cytometry and the cell cycle was analyzed with FlowJo software.

Cell apoptosis as determined by flow cytometry

The rates of cell apoptosis treated with DON were determined using the Annexin V-Alexa Fluor 647/PI Apoptosis Detection Kit (Yeason) and detected using flow cytometry. Briefly, cells were harvested at 70%-80% confluence in a 6-well plate. After washed with PBS for three times, cells were resuspended in 100 μL 1×binding buffer, followed by staining with 5 μL of Annexin V-Alexa Fluor 647 solution and 10 μL of PI solution for 15 min at room temperature in the darkness. Cells were added with 400

μL 1×Binding Buffer on ice and subjected to the flow cytometry in 1 hour. Flow cytometry was performed on a Cytoflex LX (Beckman Coulter) and the apoptosis rates were analyzed using FlowJo software (v10.4.0, BD Biosciences).

Colony formation assay

HCT116 $p53^{+/+}$ and HCT116 $p53^{-/-}$ cells were seeded into 24-well plates with 1000 cells per well, and cultured in complete DMEM media for 5 days, followed by treatment with vehicle (DMSO) or DON for 48 h. Cells were then fixed with 4% paraformaldehyde and stained with 0.1% crystal violet solution (Beyotime) for 10 min. Cells were washed with PBS twice and then imaged. The relative colony intensity was quantified with the Image J software.

Animal experiment

All animal procedures were approved by the Animal Ethics Committee of Fudan University (IDM2024024). Male BALB/c-nu nude mice (5–6 weeks old) were purchased from GemPharmatech Co., Ltd. and housed under specific pathogen-free (SPF) conditions at the Institute of Developmental Biology and Molecular Medicine of Fudan University. HCT116 cells were cultured, harvested at logarithmic growth phase, and resuspended in PBS at 5×10^6 cells/100 μL. Cell suspensions were mixed 1:1 with Matrigel and kept on ice. 200 μL cell suspension was subcutaneously injected into the axillary region of each mouse using a sterile syringe. Mice were randomly assigned to experimental groups. Tumor-bearing mice (confirmed by palpable masses at 1week post-injection) received DON (5 mg/kg) via tail vein injection every 3 days for 2 weeks. Mice were euthanized when tumors reached <2 cm^3. Tumors were excised, rinsed with saline, measured, weighed, and photographed. Tumor volume was calculated as ($width^2$ × length)/2.

Quantification and statistical analysis

Quantitative data are presented as mean ± SD. Data and images were processed and analyzed using GraphPad Prism 9. For normally distributed data between two groups,

an unpaired two-tailed Student’s t-test was used to determine significance. Log-rank test was used to determine the significance between the overall survival. p values below 0.05 were considered statistically significant; ns, not significant; * $p < 0.05$; ** $p < 0.01$; *** $p < 0.001$; **** $p < 0.0001$.

Extended results and discussion

Secondary activation by DON of the p53 pathway is mediated through the upregulation of Ser15 phosphorylation

As the phosphorylation of Ser15 of p53 is responsible for cell cycle arrest by stabilizing the protein under the conditions of glutamine deprivation [4,5], consistent with our findings that Ser15 phosphorylation of p53 significantly increased after DON treatment (Supplementary information, Fig. S2h). To determine whether Ser15 phosphorylation of p53 is the primary event induced by DON treatment, we generated a TPC1 cell line with endogenous mutant p53 replaced from Ser15 to Ala15. Notably, the p53 in S15A cells was activated by DON treatment, despite the absence of S15 phosphorylation (Supplementary information, Fig. S2i, j). On the other hand, increased phosphorylation levels result in p53 carrying more negative charges, while DON treatment shifts the charge of p53 towards a more positive state (Supplementary information, Fig. S3a). These data indicated that DON activates p53 pathway through the upregulation of Ser15 phosphorylation, although this is not believed to be the principle means of p53 pathway activation.

Deamidation alters the protein distribution of p53

As reported, p53 mutants often create aggregations that undermine its tumor-suppressive functions [1,6]. Interestingly, in TPC1 cells, CAD strongly induced p53 aggregates that appeared as puncta (Supplementary information, Fig. S3l). Immunofluorescence assays revealed increased cytoplasmic localization of deamidated p53 at N235 and N239, particularly, puncta signals were observed in HeLa cells transfected with N235D mutation plasmids, which was consistent with that observed in CAD-overexpressing cells (Supplementary information, Fig. S6c, d). These data suggested that deamidation alters the distribution of p53, which may contribute to the its malfunction.

The post-translational modification (PTM) network of p53 is multifaceted and dynamic. The potential crosstalk between N235/239 deamidation and other PTM regulatory axes remains an important question for further investigation in the future.

Discussion

Conventionally, CAD was considered as a key metabolic enzyme for pyrimidine synthesis, relying on glutamine as the primary material resource. Inhibition of CAD caused cell cycle arrest, mainly resulting from the pyrimidine deficiency[7-9]. Our study revealed that DON, a glutamine antagonist and an inhibitor of CAD, induced cell cycle arrest through the significantly activated p53 signaling pathway. Enhanced CAD activity resulted in increased p53 deamidation, which weakened p53's transcriptional regulation of the downstream target gene p21, and ultimately caused a shift in the cell cycle from the G1 to the S phase; Conversely, when DON treatment inhibited CAD activity, the level of p53 deamidation decreased, its transcriptional activity increased, and the cell cycle was arrested in the G1 phase. Our findings highlighted the signal transduction function of the metabolic enzyme CAD through p53 deamidation. We also revealed deamidation as a new post-translational modification of p53, essential for regulating the function of p53 and contributing to cell cycle regulation.

Our research broadens the understanding of DON mechanisms and target identification, which may provide with more theoretical support for the future clinical utilization of this drug. As a broad-spectrum inhibitor of the glutamine pathway, DON and its derivatives have been extensively researched and are highly anticipated in tumor therapy [10,11]. Previous studies have mainly focused on the role of DON as a glutamine blocker. However, our findings indicated that DON may produce better therapeutic effects on tumor types with wild-type p53 and high CAD expression, as it also exerts a

strong induction effect on p53 activity. Amplification of CAD genes has been reported in various human tumors [12,13]. This study suggests that CAD amplification promote tumor development by catalyzing the deamidation of p53. Consequently, the CAD copy number could serve as a diagnostic indicator for DON therapy.

The question remains open whether there are other substrate proteins of CAD that function differently with the regulated deamidation. These studies will help us understand how cells rapidly respond to metabolic enzymes activated by nutritional status. From another perspective, it is also crucial to explore how p53, as one of the most important cell guardians, directly responds to the regulation of metabolites. Low levels of the glycolytic metabolite 3-phosphoglycerate (3-PGA) serves as a signal switch to control cell fate via the post-translational modification activated p53 [14]. As cells necessitate substantial macromolecule synthesis during proliferation, the potential existence and influence of other substrates on cell cycle regulation within these biosynthesis pathways are worthy of further investigation.

Reference:

1 Chen, S. *et al.* Arsenic Trioxide Rescues Structural p53 Mutations through a Cryptic Allosteric Site. *Cancer Cell* **39**, 225-239.e228 (2021). https://doi.org:10.1016/j.ccell.2020.11.013

2 Aspinall-O'Dea, M. *et al.* Antibody-based detection of protein phosphorylation status to track the efficacy of novel therapies using nanogram protein quantities from stem cells and cell lines. *Nature Protocols* **10**, 149-168 (2014). https://doi.org:10.1038/nprot.2015.007

3 Yuan, M. *et al.* Ex vivo and in vivo stable isotope labelling of central carbon metabolism and related pathways with analysis by LC-MS/MS. *Nat Protoc* **14**, 313-330 (2019). https://doi.org:10.1038/s41596-018-0102-x

4 Lowman, X. H. *et al.* p53 Promotes Cancer Cell Adaptation to Glutamine Deprivation by Upregulating Slc7a3 to Increase Arginine Uptake. *Cell Reports* **26**, 3051-3060.e3054 (2019). https://doi.org:10.1016/j.celrep.2019.02.037

5 Reid, Michael A. *et al.* The B55α Subunit of PP2A Drives a p53-Dependent Metabolic Adaptation to Glutamine Deprivation. *Molecular Cell* **50**, 200-211 (2013). https://doi.org:10.1016/j.molcel.2013.02.008

6 Costa, D. C. F. *et al.* Aggregation and Prion-Like Properties of Misfolded Tumor Suppressors: Is Cancer a Prion Disease? *Cold Spring Harbor Perspectives in Biology* **8** (2016). https://doi.org:10.1101/cshperspect.a023614

7 Del Cano-Ochoa, F. & Ramon-Maiques, S. Deciphering CAD: Structure and function of a mega-enzymatic pyrimidine factory in health and disease. *Protein Sci* **30**, 1995-2008 (2021). https://doi.org:10.1002/pro.4158

8 Quemeneur, L. *et al.* Differential control of cell cycle, proliferation, and survival of primary T lymphocytes by purine and pyrimidine nucleotides. *J Immunol* **170**, 4986-4995 (2003). https://doi.org:10.4049/jimmunol.170.10.4986

9 Seidita, G., Polizzi, D., Costanzo, G., Costa, S. & Di Leonardo, A. Differential gene expression in p53-mediated G(1) arrest of human fibroblasts after gamma-irradiation or N-phosphoacetyl-L-aspartate treatment. *Carcinogenesis* **21**, 2203-2210 (2000). https://doi.org:10.1093/carcin/21.12.2203

10 Encarnación-Rosado, J. *et al.* Targeting pancreatic cancer metabolic dependencies through glutamine antagonism. *Nature Cancer* (2023). https://doi.org:10.1038/s43018-023-00647-3

11 Recouvreux, M. V. *et al.* Glutamine mimicry suppresses tumor progression through asparagine metabolism in pancreatic ductal adenocarcinoma. *Nature Cancer* (2023). https://doi.org:10.1038/s43018-023-00649-1

12 Ford, M. & Fried, M. Large inverted duplications are associated with gene amplification. *Cell* **45**, 425-430 (1986). https://doi.org:10.1016/0092-8674(86)90328-4

13 Chen, S., Bigner, S. H. & Modrich, P. High rate of CAD gene amplification in human cells deficient in MLH1 or MSH6. *Proc Natl Acad Sci U S A* **98**, 13802-13807 (2001). https://doi.org:10.1073/pnas.241508098

14 Wu, Y. Q. *et al.* Low glucose metabolite 3-phosphoglycerate switches PHGDH from serine synthesis to p53 activation to control cell fate. *Cell Res* (2023). https://doi.org:10.1038/s41422-023-00874-4

15 Aspinall-O'Dea, M. et al. Antibody-based detection of protein phosphorylation status to track the efficacy of novel therapies using nanogram protein quantities from stem cells and cell lines. Nature Protocols 10, 149-168 (2014). https://doi.org:10.1038/nprot.2015.007

Supplementary information, Fig. S1

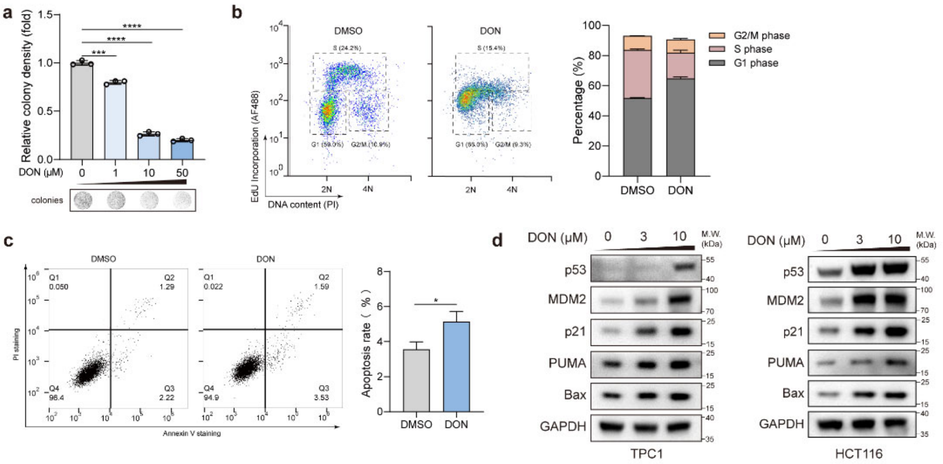

Supplementary information, Fig. S1: DON activates p53 signaling.

a Colony formation assay of HCT116 cells treated with DMSO or different doses of DON. Data are mean±SD, n = 3, with *P* values calculated by one-way ANOVA.

b Cell cycle with EdU incorporation analysis by flow cytometry in HCT116 cells treated with DMSO or DON (10 μM) for 24 h. Data are mean±SD, n = 3, with *P* values calculated by Student’ s t-test.

c Flow cytometry detection of apoptosis rate in HCT116 cells treated with DMSO or DON (10 μM) for 24 h. Data are mean±SD, n = 3, with *P* values calculated by Student’ s t-test.

d Western blot analysis of the expression of p53 and its targeted genes in TPC1 and HCT116 cells.

*$P < 0.05$; ** $P < 0.01$; *** $P < 0.001$; **** $P < 0.0001$; ns stands for no significant change.

Supplementary information, Fig. S2

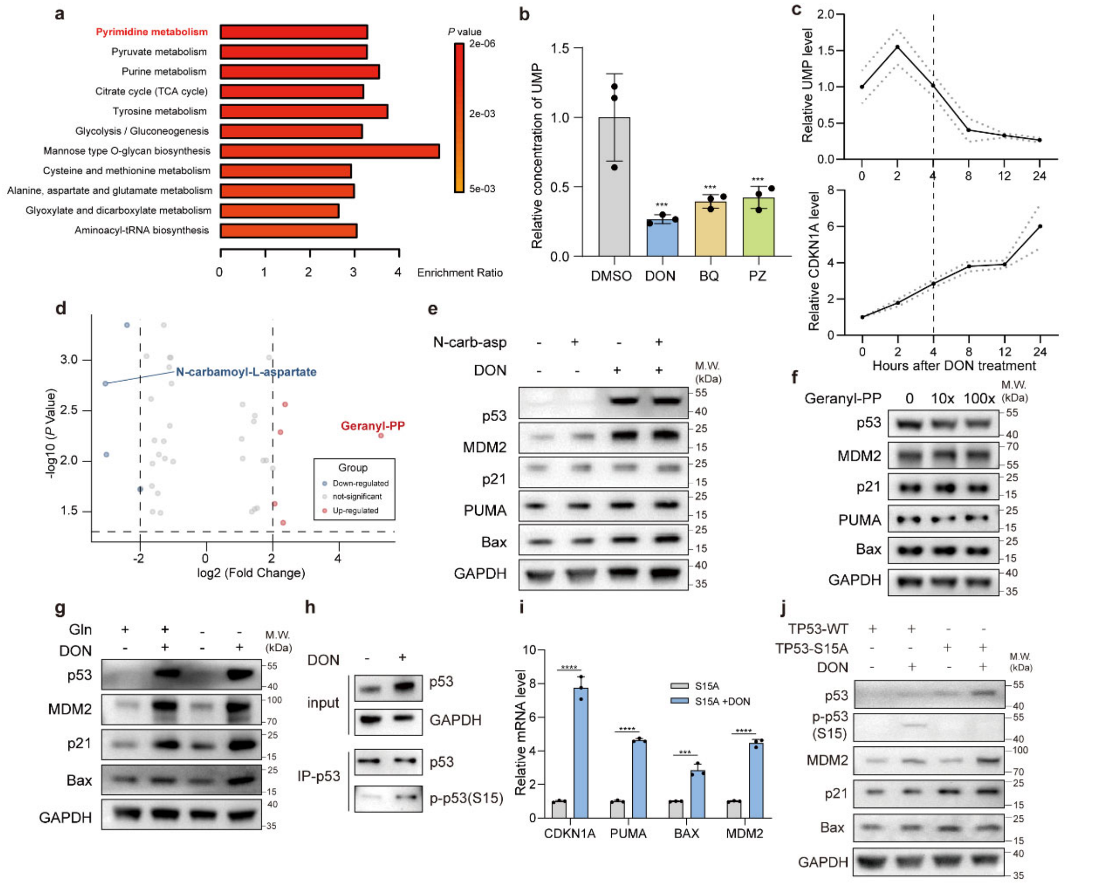

Supplementary information, Fig. S2: Significant alterations of metabolites after DON treatment inefficiently activate p53.

a Enrichment analysis of differential metabolites in HCT116 cells treated with DMSO or DON (10 μM) for 24 h.

b Relative concentration of UMP in HEK293 cells treated with DMSO, DON (10 μM), BQ (10 μM) and PZ (10 μM) for 12 h. Data are mean±SD, n = 3, with *P* values calculated by one-way ANOVA.

c Relative concentration of UMP (up) and relative mRNA levels of CDKN1A in HEK293 cells treated with DON at indicated time.

d Volcano plot of significantly differentially altered (|Fold Change|>2, p value <0.05) metabolites in HCT116 cells treated with DMSO or DON (10 μM) for 24 h.

e Western blot analysis of the expression of p53 and its targeted genes in HCT116 cells supplemented with N-carbamoyl-L-aspartate (10 μM) in the presence or absence of DON (10 μM).

f Western blot analysis of the expression of p53 and its targeted genes in HCT116 cells treated with excess concentration (100×: 45 μM; 1000×: 450 μM) of Geranyl-PP compared to physiological concentration.

g Western blot analysis of the expression of p53 and its targeted genes in TPC1 cells treated with DON (10 μM) in the presence or absence of glutamine in medium for 24 h.

h Immunoblot analysis of anti-p53 immunoprecipitates (IP line) and cell lysates (input line) from HCT116 cells treated with or without DON (10 μM) for 24 h.

i Quantitative real-time PCR analysis of mRNA levels of the indicated genes in p53-S15A endogenously mutated TPC1 cells treated with vehicle (DMSO) or JHU395 (10 μM). Data are mean±SD, n = 3, with *P* values calculated by Student' s t-test.

j Western blot analysis of the expression of the indicated genes in p53-S15A endogenously mutated TPC1 cells treated with vehicle (DMSO) or JHU395 (10 μM).

*** $P < 0.001$; **** $P < 0.0001$; ns stands for no significant change.

Supplementary information, Fig. S3

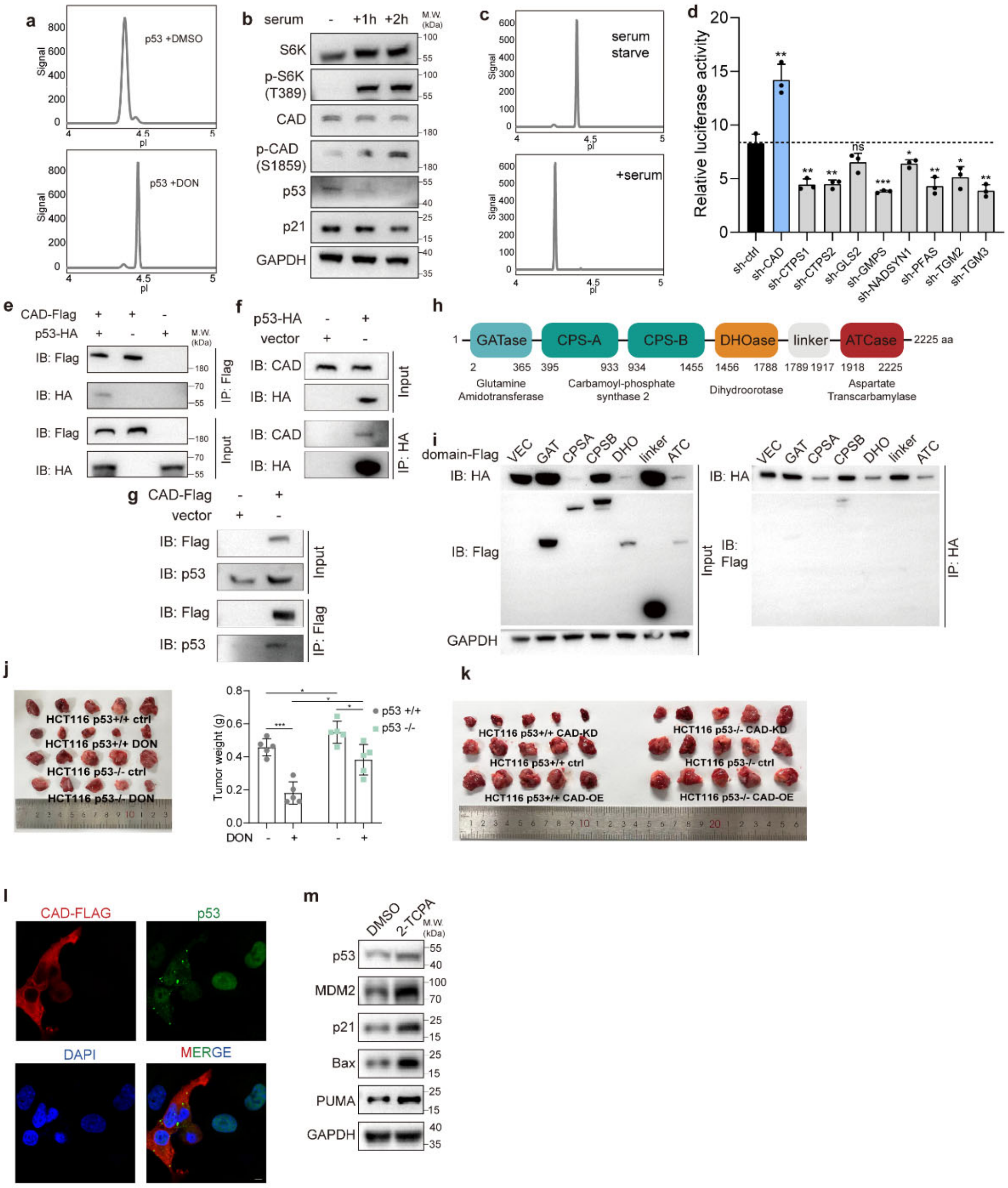

Supplementary information, Fig. S3: CAD deamidates p53.

a Capillary electrophoresis-based charge separation and immunoblot of p53 in HEK293 cells under DMSO or DON (10 μM) treatment for 24 h.

b Western blot analysis of whole cell lysates (WCLs) in TPC1 cells with serum starvation or stimulated with serum for 1 h and 2 h.

c Capillary electrophoresis-based charge separation and immunoblot of p53 in HEK293 cells with serum starvation or stimulated with serum.

d Dual luciferase reporter assay of p53 from HCT116 cells with shRNA targeting indicated cellular glutamine amidotransferases upon lenti-virus infection. CAD: Carbamoyl-Phosphate Synthetase 2, Aspartate Transcarbamylase, and Dihydroorotase; CTPS: CTP synthetase; PFAS: phosphoribosylformylglycinamidine synthetase; GMPS: GMP synthetase; NADSYN1: NAD synthetase 1; GLS2: Glutaminase 2; TGM: Transglutaminase. Data are mean±SD, n = 3, with *P* values calculated by one-way ANOVA.

e Immunoblot analysis of Flag immunoprecipitates (IP line) and cell lysates (input line) from HCT116 cells transfected with the expression vector of p53-HA and CAD-flag.

f Immunoblot analysis of CAD immunoprecipitates (IP line) and cell lysates (input line) from HEK293 cells transfected with the expression vector CAD-flag.

g Immunoblot analysis of p53 immunoprecipitates (IP line) and cell lysates (input line) from HEK293 cells transfected with the expression vector p53-HA.

h Schematic diagram showing the composition of domains within CAD protein.

i Western blot analysis of immunoprecipitates (IP line) of domains of CAD (IP line) and cell lysates (input line) from HEK293 cells transfected with the p53-HA and CAD- domain-flag.

j Xenograft models showing the *in vivo* tumor growth injected with HCT116 cells with or without p53. Data are mean±SD, n = 3, with *P* values calculated by two-way ANOVA.

k Xenograft models showing the *in vivo* tumor growth injected with HCT116 cells with or without p53.

l Representative immunofluorescence images of TPC1 cells transfected with CAD-flag plasmids, showing p53 distribution and puncta in the presence of CAD overexpression. Scale bars, 5 μm.

m Western blot analysis of the expression of p53 and its targeted genes in HCT116 cells treated with 2-TCPA (20 μM) for 24 h.

*$P < 0.05$; ** $P < 0.01$; *** $P < 0.001$; ns stands for no significant change.

Supplementary information, Fig. S4

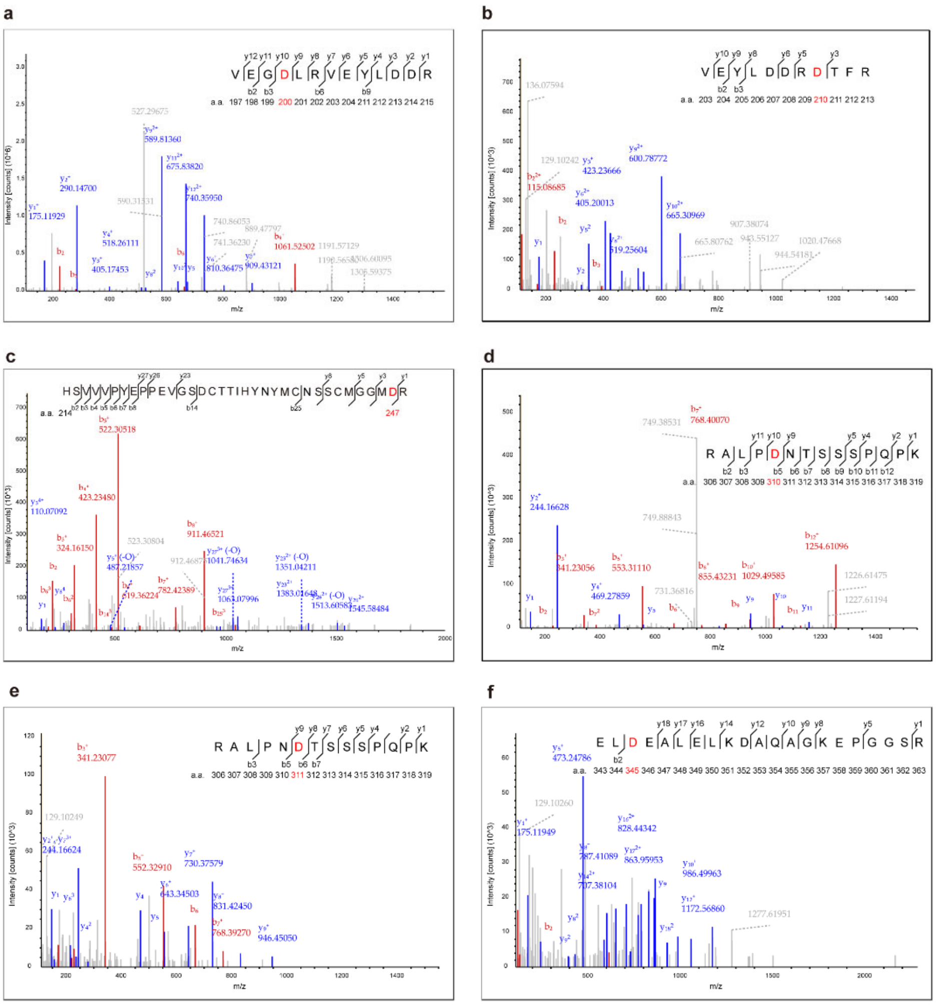

Supplementary information, Fig. S4: Deamidation residues within p53.

a-f The m/z spectra of the peptides containing (a) N200D, (b) N210D, (c) N247D, (d) N310D (e) N311D and (f) N345D are shown, with D marked in red due to deamidation, of the p53-flag protein purified with flag-tagged magnetic beads from HCT116 cells.

Supplementary information, Fig. S5

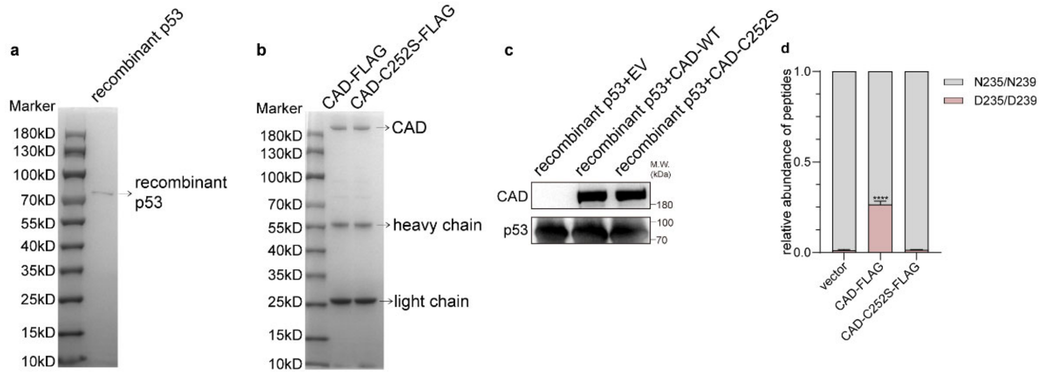

Supplementary information, Fig. S5: In vitro deamidation assay of p53 by CAD

a Coomassie-stained SDS-PAGE (4-15% gel) showing N-terminal 6×His-SUMO-tagged human p53 (CSB-EP024077HU, CUSABIO). Arrow indicates full-length p53. Molecular weight markers (kDa) shown on left.

b Coomassie staining of FLAG affinity-purified proteins from 293T lysates: lane 1 (WT CAD-FLAG), lane 2 (C252S CAD-FLAG). Predicted CAD molecular weight marked by arrowhead. Molecular weight markers (kDa) shown on left.

c Western blot analysis of reaction mixtures after in vitro deamidation assay using anti-p53 (9282, CST) and anti-CAD (11933, CST) antibodies.

d Histogram displays relative abundance of deamidated (pink) vs native (gray) peptides from three independent experiments (mean ± SD, n=3). Statistical significance (*****P*<0.0001).

Supplementary information, Fig. S6

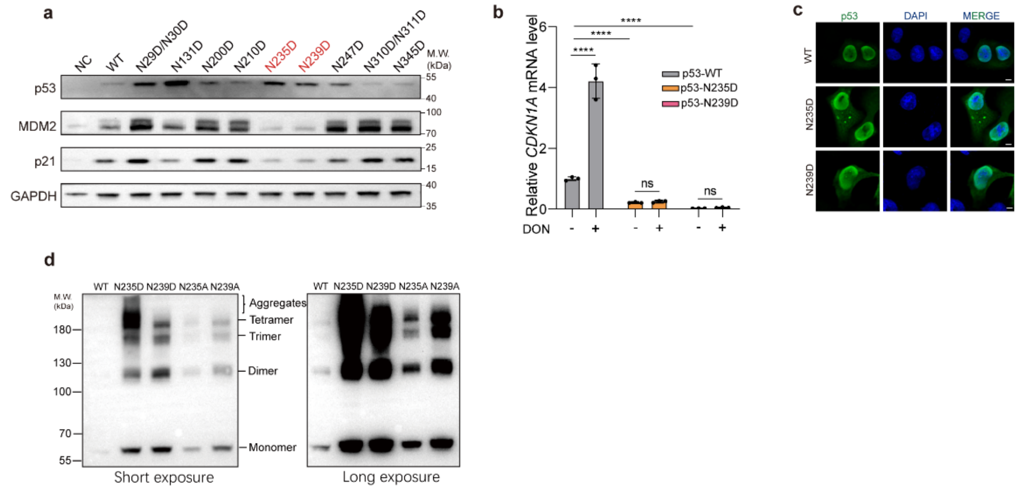

Supplementary information, Fig. S6: Deamidation at the conserved N235 or N239 residue of p53 impairs its transcriptional activity.

a Western blot analysis of whole cell lysates from HeLa cells transfected with the indicated mutant p53-flag plasmids.

b Quantitative real-time PCR analysis of relative mRNA levels of CDKN1A in wild-type and p53-N235D, p53-N239D endogenously mutant TPC1 cells treated with DMSO or DON (10 μM) for 24 h. Data are mean±SD, n = 3, with P values calculated by two-way ANOVA. *$P < 0.05$; ** $P < 0.01$; *** $P < 0.001$; **** $P < 0.0001$; ns stands for no significant change.

c Representative immunofluorescence images of HeLa cells transfected with p53-WT, p53-N235D and p53-N239D plasmids, showing the distribution and puncta of p53. Scale bars, 5μm.

d HeLa cells were transfected with p53-WT, p53-N235D, p53-N235A, p53-N239D and p53-N239A plasmids and fixed with 0.01% (v/v) glutaraldehyde (GA), followed by immunoblotting.

Supplementary information, Fig. S7

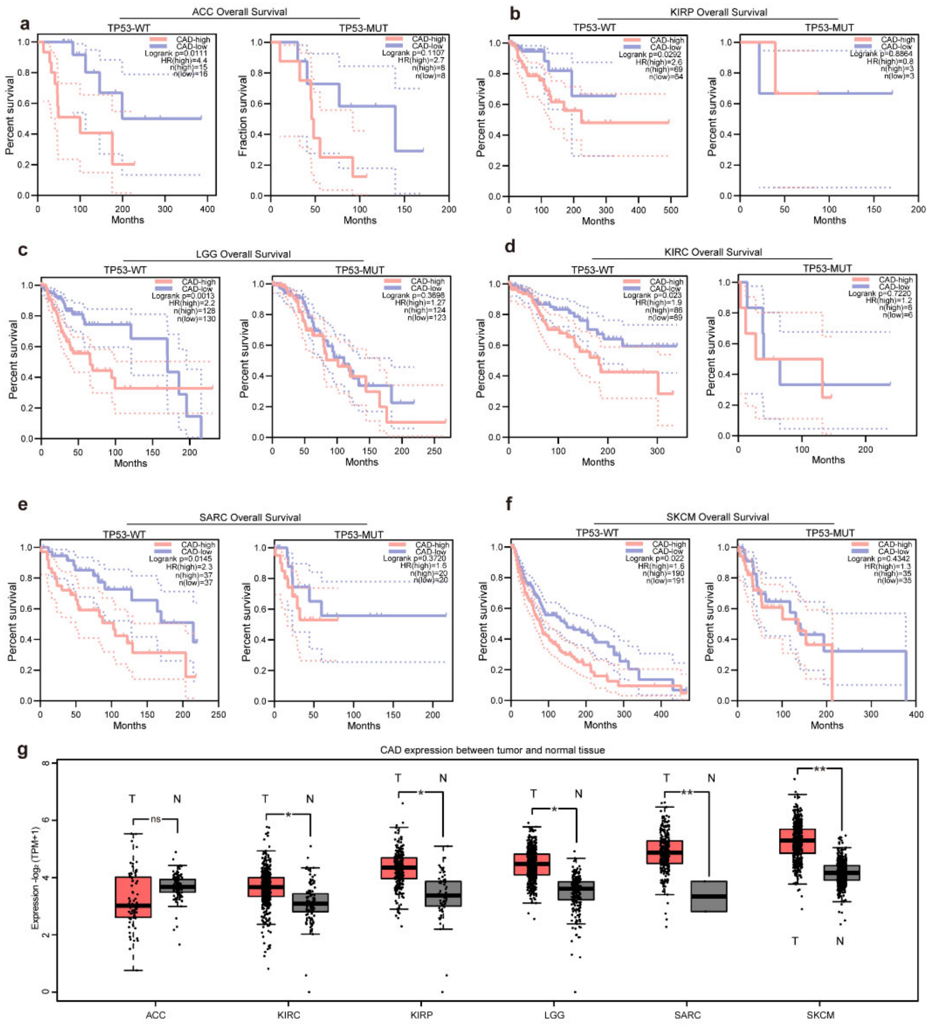

Supplementary information, Fig. S7: Survival analysis of CAD expression between different TP53 status in different tumor types.

a-f Analysis of different tumor types, including adrenocortical carcinoma (ACC), kidney renal clear cell carcinoma (KIRC), kidney renal papillary cell carcinoma (KIRP), Brain Lower Grade Glioma (LGG), Sarcoma (SARC) and Skin Cutaneous Melanoma (SKCM), comparing the overall survival curves between patients carrying wild-type p53 (left panel) with those carrying mutant or null p53 (right panel) with high and low CAD expression. *P* values calculated by log rank t-test.

g Analysis of CAD expression levels between tumor tissue and normal tissues in different cancer types in accordance with S4a-f. *P* value was calculated by Welch’ s t-test. *$P < 0.05$; ** $P < 0.01$; ns stands for no significant change.

Supplementary information, Table. S1

Name	Sequence (5'-3')
CDKN1A-qPCR-F	GTCTTGTACCCTTGTGCCTC
CDKN1A-qPCR-R	CAAAATAGCCACCAGCCTCTTCT
PUMA-qPCR-F	ACGACCTCAACGCACAGTACG
PUMA-qPCR-R	TCCCATGATGAGATTGTACAGGAC
MDM2-qPCR-F	ATCAGGCAGGGGAGAGTGAT
MDM2-qPCR-R	CAATTCTCACGAAGGGCCCA
BAX-qPCR-F	GATGCGTCCACCAAGAAGCT
BAX-qPCR-R	CGGCCCCAGTTGAAGTTG
TP53-qPCR-F	GAGGTTGGCTCTGACTGTACC
TP53-qPCR-R	TCCGTCCCAGTAGATTACCAC
GAPDH-qPCR-F	GAAGGTGAAGGTCGGAGTC
GAPDH-qPCR-R	GAAGATGGTGATGGGATTTC
Human sh-ASNS	GCTGTATGTTCAGAAGCTAAA
Human sh-CTPS	GCTCTCACATTACCTCCAGAA
Human sh-CTPS2	GCATTGGTAAAGGGATCATTG
Human sh-GFPT1	CGTCTTTCTATCCATCGAATT
Human sh-GFPT2	ATCCGTGGCTTGAGATCTTTA
Human sh-GMPS	GAACAACAACCCTACCAATAT
Human sh-NADSYN1	AGGTTTGATCTGCGACCATTT
Human sh-PPAT	GTAGCTTCACCACCAATTAAA
Human sh-GLS2	CGGAATTATGCCATCGGCTAT
Human sh-PFAS	GCCAGGCATGGAAGTTGTAAA
Human sh-TGM2	TGAGAAATACCGTGACTGCCT
Human sh-TGM3	GAGGCAGAACATCCCATAAAG
Human sh-CAD-1	GCTCCGAAAGATGGGATATAA
Human sh-CAD-2	CGAATCCAGAAGGAACGATTT
Human sh-CAD-3	CCCAGATGAAATGGATGAGTT
CAD-sgRNA-KO-1	ACCTGTCTTTGGGATCTGCCTGG
CAD-sgRNA-KO-2	GAACGGCATGTACATCCGCATGG
TP53-sgRNA-KO	AGCACATGACGGAGGTTGTG
TP53-sgRNA-S15A	CGTCGAGCCCCCTCTGAGTC
TP53-sgRNA-N235N239	TGTTACACATGTAGTTGTAG
